# New improved incision-tubing approach for bronchoesophageal Fistula with mediastinal abscess after esophagectomy: A case report

**DOI:** 10.3389/fsurg.2023.1100264

**Published:** 2023-03-07

**Authors:** Jianwei Cao, Mingfei Geng, Xiaoyu Huang, Changjiang Liu, Hui Li

**Affiliations:** ^1^ Department of Thoracic Surgery, Beijing Chaoyang Hospital, Capital Medical University, Beijing, China; ^2^ Department of Thoracic Surgery, Anyang Tumor Hospital, The Fourth Affiliated Hospital of Henan University of Science and Technology, Anyang, China; ^3^ Department of Thoracic Surgery, The Fuorth Hospital of Hebei Medical University, Shijiazhuang, China

**Keywords:** esophagectomy, bronchoesophageal fistula, minimally invasive management, esophageal cancer, gastric conduit

## Abstract

Bronchoesophageal fistula is a serious threat to the survival after esophagectomy for esophageal cancer. The erosion of mediastinal abscess post anastomotic leakage is the most likely directly cause. However, the bronchoesophageal fistula with gastric conduit necrosis and mediastinal abscess is refractory to either surgical or conservative treatment. In the article, a unique case of Bronchoesophageal fistula with mediastinal abscess after gastric conduit necrosis is presented. A 74-year-old female was detected the right inferior bronchus-esophageal fistula with mediastinal abscess on 15 postoperative day after esophagectomy for esophageal cancer. A successful new improved minimally invasive management was performed.

## Introduction

Bronchoesophageal fistula (BEF) post esophagectomy for esophageal cancer is a rare but fatal complication (1). The treatment of BEF is intricate and no unified cognation. When BEF due to gastric conduit necrosis and subsequent severe mediastinal abscess, the treatment become more difficult and challengeable, and conservative therapy seems impossible. We present herein a particularly rare case, a fistula which due to mediastinal abscess after gastric conduit necrosis from right lower lobar bronchus to cervical esophageal anastomosis, that we treated with successful minimally invasive management.

## Case report

A 74-year-old female was admitted to Anyang Tumor Hospital with a one month history of progressive dysphagia. Clinical T2N0M0 stage (2) squamous cell carcinoma was diagnosed by upper gastrointestinal endoscopic ultrasonography and computed tomography (CT). Physical examination and laboratory data didn’t reveal abnormal findings.

**Figure 1:**
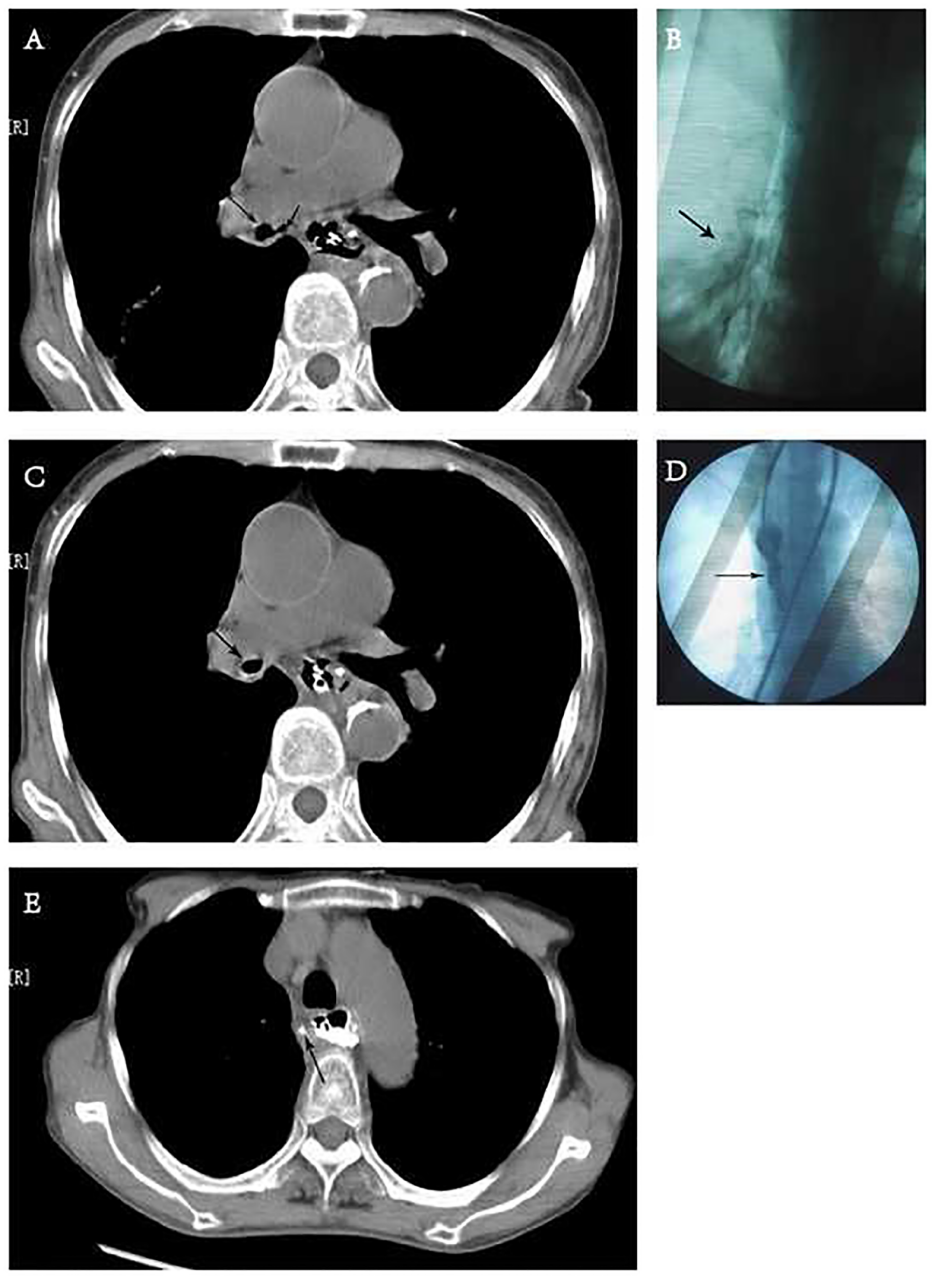
Chest computed tomography (CT) and fluoroscopy. (**A**) CT showed the bronchus communicate with mediastinal abscess. Long arrow: right inferior bronchus. Short arrow: mediastinum. (**B**) Dimeglumine flowed into right low lobe *via* mediastinal drainage tube through right inferior bronchus in chest fluoroscopy. Arrow: dimeglumine. (**C,D**) CT and fluoroscopy (pulled dimeglumine *via* mediastinal drainage tube) showed the fistula of bronchus is healed. (**E**) CT showed mediastinal abscess was healed after oral dimeglumine. Arrow: sinus tract after removing mediastinal drainage tube.

**Figure 2:**
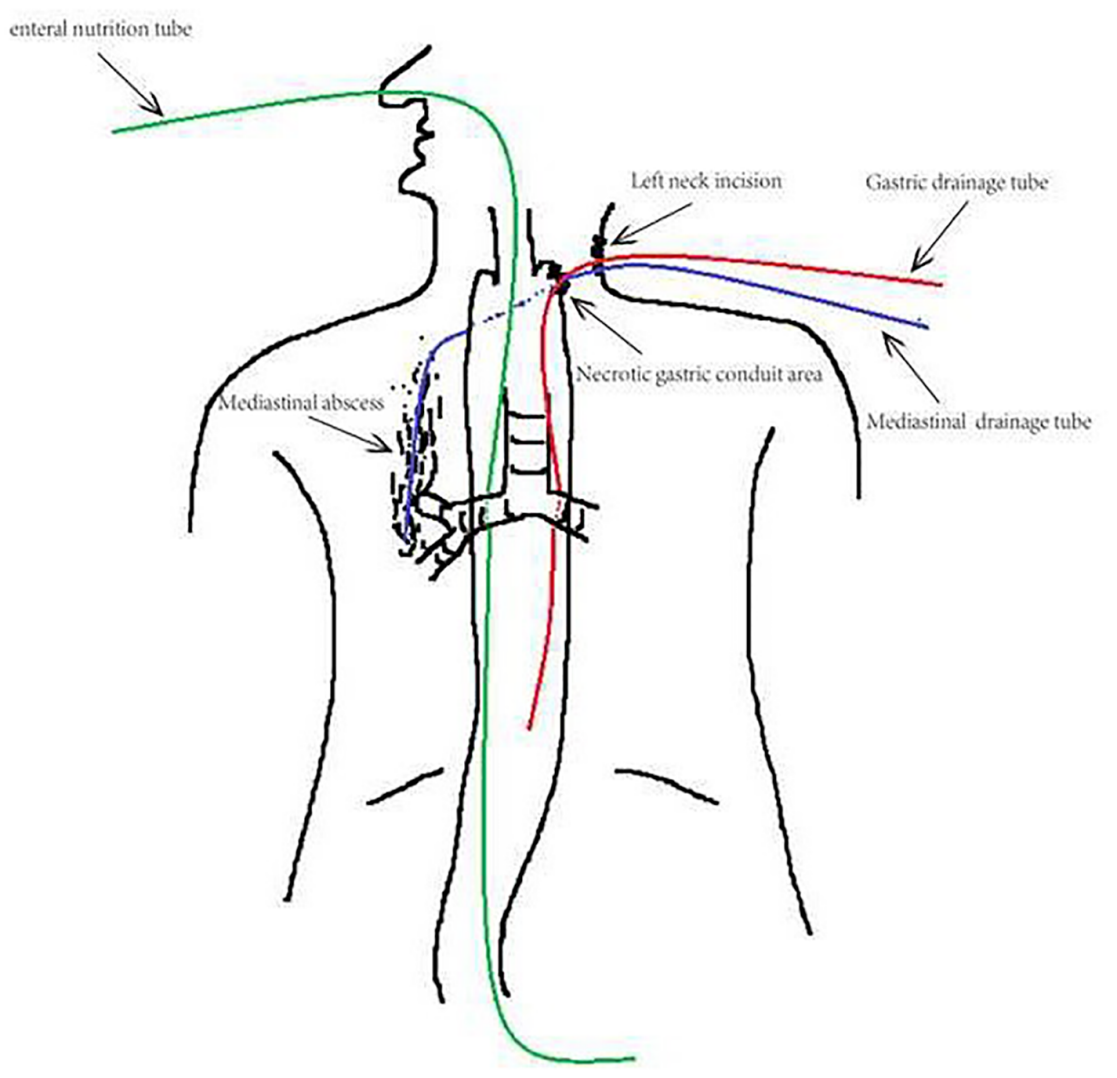
Insertion of drainage tube. Feeding tube was inserted into duodenum intraoperation *via* nose to afford enteral nutrition. Gastric and mediastinal drainage tubes were along primary neck incision, and both continuously decompressed. The bottom of mediastinal drainage tube was obtuse and obstructed, had 4 side holes, and 1 cm distant from the right inferior bronchus.

The patient underwent esophagectomy and lymphadenectomy *via* open left thoracotomy. Esophagus was replaced by gastric conduit through posterior mediastinum, with circular- stapled end-to-side esphagogastric anastomosis on left neck. Gastric conduit was constructed along the greater curvature of the stomach using linear staplers. All of the vessels were dissected except for the right gastroepiploic vessels and a few branches of the right gastric vessels, and the width of the gastric conduit was confined to 4 cm. Naso-gastric decompression tube and naso-duodenal feeding tube were placed in operation. White blood count (WBC) was normal and patient incautiously removed naso-gastric decompression tube on postoperative day 5 (POD5). Obviously anastomotic leakage was confirmed by chest fluoroscopy (oral dimeglumine) on POD7 and WBC was 22.6*109/l. Then, opened the neck incision, partly gastric conduit stump is pallid with cacosmia, and saliva outflowed from stump when patient swallowed. We cut out necrotic organization (1 cm*1 cm), inserted gastric decompression tube *via* this gastric conduit incision, and placed drainage strip to anastomosis through neck incision. In the same time, antibiotics was re-granted with intensive par- and enteral nutrition. Follow 8 days, there was intermittent fever. On POD15, patient suddenly occurred fierce cough and brown fetid sputum. CT and chest fluoroscopy (dimeglumine) confirmed mediastinal abscess and right lower lobar bronchus fistula (Figure 1A,B). Chest fluoroscopy-guided mediastinal continuous decompression drainage tube (gastric drainage tube, 5.33 mm, Yangsheng Medical Science, Yangzhou, Jiangsu, China) was inserted along gastric conduit through cervical incision (Figure 2), and 100 ml brown fetid thick pus was extracted. Temperature and WBC recovered soon afterwards, and the cough gradually improved. 6 days later, BEF was healed (Figure 1C,D), and then mediastinal decompression drainage tube was gradually intermittent extracted.

Gastric and mediastinal drainage tubes were removed after the healing of anastomotic leakage (confirmed by oral dimeglumine fluoroscopy on POD31) (Figure 1E), and the neck incision was closed with adhesive tape immediately. The patient was discharged after 3days no discomfort when taking liquid diet. The diet recovered well after 2 weeks, and there was no anastomotic stricture and recurrent leakage in the eight months follow up.

## Comment

Though the incidence is as low as 0.28% to 3%, BEF is a fatal complication, and gastric conduit necrosis and anastomotic leakage are the most common reasons for it (3, 4). Inflammatory erosion due to mediastinal abscess for adjacent bronchus is the immediate mechanism.

There is still no standard for the treatment of BEF, and lack of large series research. In previous reports, surgical management and endoscopic stent insertion were the main treatment, and a few case reports mentioned conservative management. Balakrishnan A et al. reported a more samples (11 patients) retrospective review about surgical repair of BEF (3). The operations included repairing fistula, resecting organ, and reconstructing esophagus. Although most cases were effective, postoperative mortality was high (27.3%), and only 3 patients resumed an oral diet in their report. In one study with 7 patients BEF induced by anastomotic leakage, endoscopic stent insertion was recommended with 75% successful samples (4). They preferred to perform surgical resection for 2 patients complicated gastric conduit necrosis, but no patient survival after 2 weeks. Bona D et al. reported one case with right inferior bronchus- esophagus fistula cured by conservative management, but they main relied on a synthetic glue, metal chip, and self-expanding plastic stent (5). Martin-Smith JD et al. made a successful totally conservative treatment with antibiotics, enteral nutrition, and non-invasive respiratory support for BEF (6). However, the patent didn't have mediastinal inflammation and severe systems. In this report, the patient already occurred syndrome of necrotizing descending mediastinitis (NDM) before BEF. NDM is a life-threatening infection, and the mortality is high if couldn't treated quickly and thoroughly. Timely and effective drainage is the key point for the treatment of NDM (7, 8). In previous reports, surgical management was recommended firstly when gastric conduit necrosis and mediastinal abscess existing. Unfortunately, the patient was 74 years old with poor condition, and she refused to endoscopy scan. We herein preferred to attempt minimally invasive management. It is like to cure anastomotic leakage with mediastinal abscess in two reports (9, 10). There were some new improvements in our report as follows: (I) difference of drainage tube insertion site. We used the original neck incision to avoid new trauma, decrease discomfort. Furthermore, larger diameter drainage tube could be inserted easier to avoid obstructed by sticky pus. (II) difference of mediastinal drainage tube. A 5.33 mm gastric drainage tube with obtuse and obstructed terminal was used inserted into abscess cavity. We made three holes along the tube side in the same time. (III) The drainage tube didn't be inserted to the bottom of abscess cavity but 1 cm distance to avoid impact for the fistula of bronchus.

There are some limitations to this report. First, mediastinal abscess was not found and dispose in time. Second, endoscopy was refused, and we only diagnosed the BRF *via* syndrome and fluoroscopy, so couldn't confirm the diameter of fistula in bronchus. Third, we didn't put a perianastomotic drain which couldn't decrease the incidence of anastomotic leakage. Perianastomotic drain can led to earlier discovery of anastomotic leakage, reduce the incidence of fatal mediastinitis and mortality from anastomotic leakage.

This case report is unique as the conservative management is rare used for the patient with severe mediastinal abscess beside BEF, and we made some new improvement like the three points. In conclusion, improved conservative management seems to be suitable for BEF with gastric conduit necrosis and mediastinal abscess. When necrosis is small, we may attempt conservative management.

## Data Availability

The original contributions presented in the study are included in the article/Supplementary Material, further inquiries can be directed to the corresponding author.
